# Elevated cerebral blood flow in distal and intermediate arterial territories in pediatric sickle cell anemia

**DOI:** 10.3389/fnins.2026.1868009

**Published:** 2026-08-05

**Authors:** Ping Zou Stinnett, Zachary Abramson, Nicholas S. Phillips, Jane S. Hankins, Andrew M. Heitzer, Akshay Sharma, Yimei Li, Tushar Patni, Ranganatha Sitaram

**Affiliations:** 1 Department of Radiology, St. Jude Children’s Research Hospital, Memphis, TN, United States; 2 Department of Psychology and Behavioral Sciences, St. Jude Children’s Research Hospital, Memphis, TN, United States; 3 Department of Global Pediatric Medicine, St. Jude Children’s Research Hospital, Memphis, TN, United States; 4 Department of Hematology, St. Jude Children’s Research Hospital, Memphis, TN, United States; 5 Department of Bone Marrow Transplantation and Cellular Therapy, St. Jude Children’s Research Hospital, Memphis, TN, United States; 6 Department of Biostatistics, St. Jude Children’s Research Hospital, Memphis, TN, United States

**Keywords:** arterial flow territory, arterial spin labeling, cerebral blood flow, cerebral hemodynamic adaptation, sickle cell anemia, transcranial Doppler

## Abstract

**Introduction:**

Children with sickle cell anemia (SCA) are at increased risk of cerebrovascular complications due to chronic anemia and progressive vascular injury, yet how their cerebral hemodynamics are altered remains poorly understood. Previous research has shown that overall cerebral blood flow (CBF) is often higher in SCA, likely as an adaptive response to their chronic anemia, while this CBF increase is also linked to a greater risk of stroke, silent infarcts, and cognitive deficits. Distinguishing when this elevation reflects effective compensation versus early pathophysiology is critical.

**Methods:**

This study examined spatial patterns of cerebral blood flow (CBF) and their relationship with transcranial Doppler (TCD) velocity to better characterize hemodynamic adaptation in pediatric SCA. CBF was quantified using arterial spin labeling MRI in 32 children with HbSS or HbS/β^0^-thalassemia (median age, 12.3 years; range, 7.2–17.8) across the anterior (ACA), middle (MCA), and posterior (PCA) cerebral artery territories, and their proximal, intermediate, and distal sub-territories in gray and white matter.

**Results:**

Across all three territories, distal and intermediate sub-territories exhibited significantly higher CBF than proximal regions (*p* < 0.0001), and these sub-territory differences increased with age (*p* ≤ 0.04). Distal CBF was significantly lower than intermediate CBF (*p* < 0.0001) in only MCA but not in PCA and ACA. Although none of the patients had abnormal TCD velocities and only five showed conditionally elevated TCD velocities in one territory, 38% showed silent cerebral infarctions. CBF and TCD velocities in corresponding territories were not significantly correlated, except in the left ACA and right MCA gray matter, where weak inverse correlations were observed.

**Conclusions:**

These findings suggest that compensatory increases in CBF in response to chronic anemia occur primarily at the microvascular level within distal and intermediate sub-territories, highlighting the vulnerability of the distal microcirculation especially in MCA. Territorial perfusion mapping may therefore reveal early hemodynamic adaptations not captured by global CBF measures or detectable by conventional TCD screening. This approach may improve early detection and support better treatment strategies in patients.

## Introduction

Sickle cell disease is a hereditary hemoglobinopathy that affects millions of people worldwide, including approximately 100,000 individuals in the United States, most of whom have African ancestry (Collaborators, 2023; Piel et al., 2023). The characteristic sickle-shaped red blood cells (RBCs), formed under hypoxic conditions, are rigid, prone to hemolysis, and can occlude small blood vessels (Sundd et al., 2019). These abnormalities lead to chronic anemia and hemodynamic alterations that affect nearly all organs, including the brain. Individuals who are homozygous for the sickle hemoglobin gene (HbSS) or co-inherit the sickle mutation with a β-thalassemia mutation (HbS/β^0^) have sickle cell anemia (SCA), the clinically more severe form of the disease. SCA is associated with significant cerebrovascular complications, including overt stroke and silent cerebral infarctions (SCI) (Wang, 2007). These neurological events can lead to long-term cognitive impairment and reduced quality of life (Wang, 2007; Ohene-Frempong et al., 1998). Individuals with SCA have decreased arterial oxygen-carrying capacity and their blood oxygenation level-dependent responses to neurostimulation were diminished (Zou et al., 2011). To maintain adequate cerebral oxygen delivery, autoregulatory mechanisms increase cerebral blood flow (CBF) (Bush et al., 2016). However, increased CBF has been associated with an increased risk of stroke (Prussien et al., 2019) and is inversely correlated with neurocognitive functioning (Strouse et al., 2006; Stotesbury et al., 2022).

Transcranial Doppler (TCD) ultrasound is a simple and universally available tool that is currently employed as a screening modality for assessing stroke risk in children with SCA (Mazzucco et al., 2017; Bulas, 2005). When the TCD velocities in major cerebral arteries, particularly the middle cerebral and internal carotid arteries, are abnormally elevated, they indicate elevated stroke risk. Regular blood transfusion therapy is recommended to reduce sickle hemoglobin concentration, increase total hemoglobin concentration, and decrease the risk of future stroke in children with abnormal TCD values (Guilliams et al., 2018).

However, TCD velocity is a surrogate marker that reflects a composite response to multiple factors, including vessel diameter, blood viscosity, anemia, and underlying vasculopathy. TCD does not directly quantify the volume of blood perfusing cerebral tissue and cannot assess microvascular or regional perfusion (Croal et al., 2017). TCD is primarily sensitive to macrovascular changes, large-vessel stenosis or narrowing, while being influenced by global CBF changes due to anemia and other systemic factors. This limitation is critical because even with normal TCD velocities, regional hypo-perfusion may still occur at brain regions at greatest risk of experiencing ischemia and stroke may also occur (Adams et al., 2004). A large-scale study has shown no associations between TCD velocity and neurocognitive outcomes in children with SCA (Longoria et al., 2022). Moreover, TCD measurements are highly operator-dependent and can be affected by skull bone thickness, which may introduce variability (Ali, 2021).

In contrast, CBF measured with non-invasive magnetic resonance imaging (MRI) techniques such as arterial spin labeling (ASL) quantifies actual tissue perfusion, providing direct insight into microvascular function and regional oxygen delivery. This distinction is particularly relevant in the context of SCA, where chronic anemia induces compensatory increases in CBF to maintain adequate oxygen delivery despite reduced hemoglobin concentration. Previous studies have shown that children with SCA exhibit elevated CBF compared to healthy controls across brain regions (Kawadler et al., 2018; Stinnett et al., 2025). Although this hyper-perfusion is thought to represent an adaptive response to anemia, it may also reflect a state of hemodynamic stress that renders the brain vulnerable to ischemic injury.

Additionally, as CBF is not uniform across the brain in healthy children (Zou et al., 2021; Avants et al., 2015), the distribution of CBF and its compensatory elevation in children with SCA are likewise nonuniform (Stinnett et al., 2025). Certain regions, particularly watershed zones between major vascular territories, may still experience inadequate perfusion even when global CBF is elevated. ASL studies have demonstrated regional hypoperfusion in individuals with untreated severe obstructive sleep apnea due to intermittent hypoxemia (Nie et al., 2017), and in healthy volunteers exposed to prolonged hypoxia at high altitude despite global increases CBF (Lawley et al., 2017). These observations underscore the limitations of using global CBF as a sole marker of cerebral tissue perfusion, given the complex and heterogeneous cerebrovascular physiology of SCA (Fields et al., 2018).

Despite the successful use of TCD screening to identify patients at risk for stroke, the relationship between TCD velocity and CBF remains poorly understood. Clarifying this relationship is essential for accurate interpretation of cerebrovascular imaging in SCA and for improving stroke risk stratification beyond what is currently performed with TCD alone. A more comprehensive understanding of how macrovascular and microvascular hemodynamic measures are related could enhance individualized treatment strategies and early detection of perfusion deficits in this high-risk population.

In this study, we aimed to characterize CBF distributions across major arterial territories and to investigate the relationship between TCD velocities and ASL-derived CBF in children with SCA. This work may enhance understanding of cerebrovascular adaptation in SCA and help inform strategies for risk stratification and disease management.

## Materials and methods

### Participants

Thirty-two children [16 females and 16 males; median age 12.3 years (range 7.2–17.8)] with a diagnosis of HbSS or HbS/β^0^-thalassemia participated in this study. Patients were excluded if they were unable to tolerate MRI without sedation or anesthesia; had a contraindication to 3 T MRI; had a positive pregnancy test at the time of MRI; had previously received hydroxyurea therapy, transfusion therapy, a stem cell transplant, or other myelosuppressive therapy; or had a history of clinical stroke. Each eligible participant underwent MRI, blood tests, neurocognitive assessment, and transcranial Doppler ultrasound (TCD). The study was approved by the St. Jude Children’s Research Hospital Institutional Review Board and conducted in accordance with the Declaration of Helsinki. Written informed consent was obtained from each participant’s guardian, and all children gave verbal assent to participate.

### MRI and image analysis

MRI data were acquired on a 3 T Siemens scanner. Anatomical sequences included sagittal 3D high-resolution T1-weighted (MPRAGE), sagittal 3D T2 FLAIR (SPACE), sagittal 3D T2-weighted (SPACE), axial 2D T1-weighted (FLASH), and axial 3D time-of-flight MRA for evaluation of morphological and vascular abnormalities, including SCI and arterial stenosis or occlusion. CBF was quantified (Zou et al., 2021; Luh et al., 1999) using the Q2TIPS pulsed ASL sequence (Luh et al., 1999). Each patient’s CBF image was co-registered to their 3D T1-weighted MPRAGE image, which was segmented into gray and white matter masks using the Unified Segmentation approach implemented in SPM12.^1^

**Figure 1:**
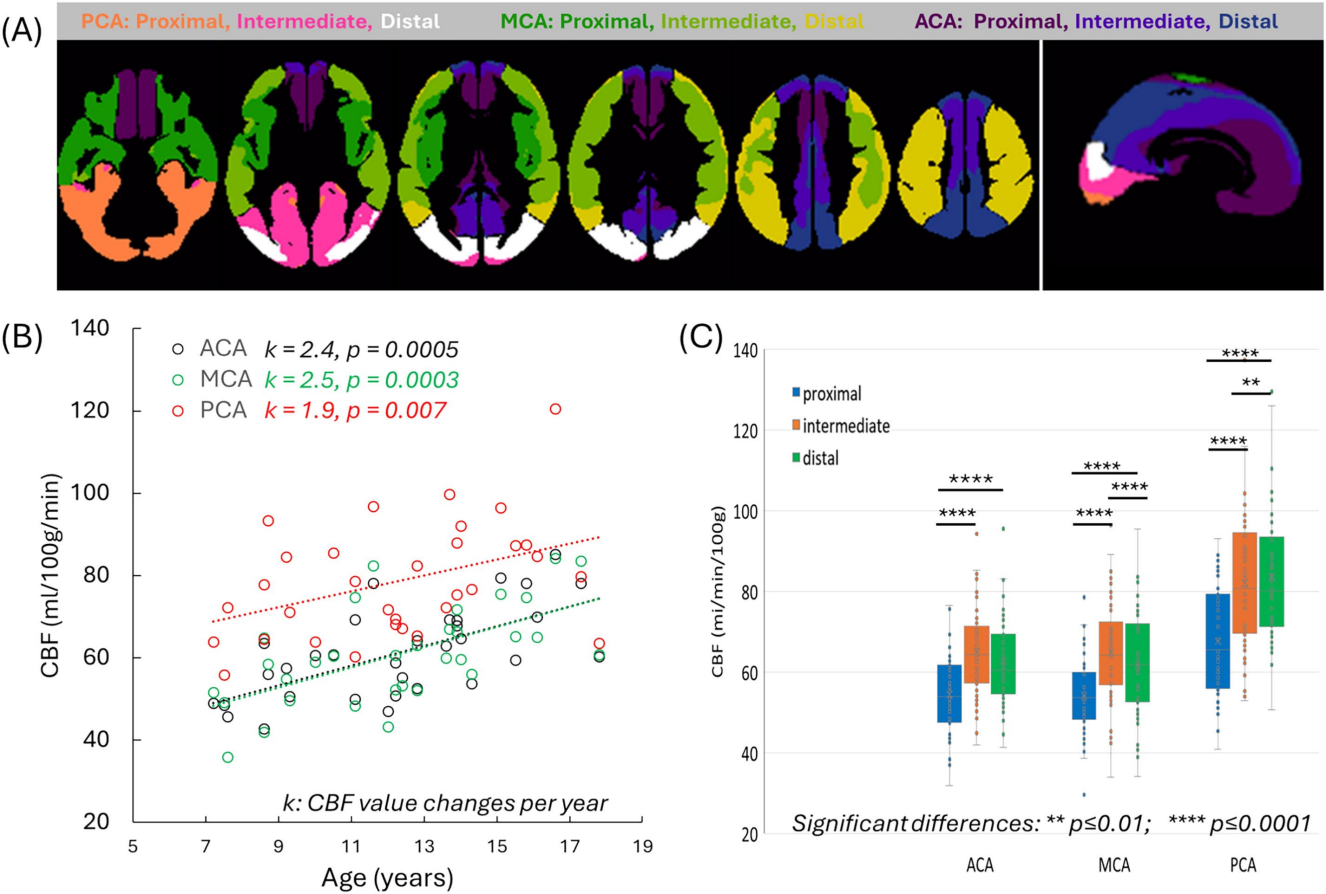
CBF in arterial flow territories. **(A)** Color-coded segmentations show the arterial flow territories of the anterior cerebral artery (ACA), middle cerebral artery (MCA), and posterior cerebral artery (PCA), each further subdivided into three sub-territories, proximal, intermediate, and distal. **(B)** Gray matter CBF increased significantly with age across the three flow territories. White matter (not shown) CBF showed a similar trend. See Table 1 for rates of CBF changes and corresponding *p*-values. **(C)** Gray matter CBF in sub-territories (proximal, intermediate, and distal) for the ACA, MCA and PCA. CBF in the PCA territory was significantly higher than in the ACA and MCA territories, and CBF values in distal and intermediate sub-territories were significantly higher than in proximal regions across all three territories. White matter (not shown) CBF had similar regional patterns. See Table 1 for *p*-values for the regional comparisons in both gray and white matter.

The standard template (Tatu et al., 1998; Mutsaerts et al., 2015; Figure 1A) included perfusion territories supplied by the bilateral anterior cerebral arteries (ACA), middle cerebral arteries (MCA), and posterior cerebral arteries (PCA). Each flow territory was further subdivided into proximal, intermediate, and distal sub-territories (Mutsaerts et al., 2015). Using the inverse normalization, these territories were aligned with each patient’s CBF map, and the gray matter and white matter CBF values were extracted from each arterial territory and sub-territory. This territory-specific mapping approach provides a means to characterize regional heterogeneity in CBF within the major cerebral arterial territories, revealing spatial patterns that may not be captured by global CBF measures.

### TCD examination

Standard non-imaging TCD were performed in the clinic by STOP II certified TCD examiners (Nichols et al., 2001). An abnormal result was defined by the STOP criteria (Bulas, 2005; Nichols et al., 2001) as a time-averaged maximum mean velocity (TAMMV) ≥ 200 cm/s in either the MCA, the distal internal carotid artery (dICA) or the ACA. A conditional result was defined as a TAMMV of 170–199 cm/s in the dICA, MCA or ACA or a velocity ≥170 cm/s in the PCA or basilar artery. An exam with TAMMV less than 170 cm/s in all measured vessels was considered normal.

The velocities from left and right ACA, MCA, and PCA were used to correlate with the corresponding territory CBF values.

### Statistical analysis

Pearson correlations were calculated between gray and white matter CBF values, between hemispheres, and between CBF and corresponding TCD velocities in the ACA, MCA, and PCA territories. Age effects on CBF within each flow territory and on sub-territory CBF differences were evaluated using linear mixed-effects models. Comparisons among territories and sub-territories were performed with one-way repeated-measures ANOVA, followed by paired t-tests when main effects were significant. *p-*values were adjusted with Bonferroni correction, and statistical significance was set at *α* = 0.05. Analyses were conducted in R (version 4.1.2).

## Results

Twenty-nine patients had HbSS and three had HbS/β^0^-thalassemia. They had below normal levels hemoglobin at median (range) 8.1(6.9, 11.1) g/dL and hematocrit at median (range) 23.4(18.8, 31.8) %. Two patients (6%) showed stenosis on MRA. Twelve (38%) patients had evidence of SCI on brain MRI. SCIs were most prominently located in the centrum semiovale, frontal subcortical white matter, and frontoparietal white matter. None of the participants were receiving hydroxyurea at the time of MRI or TCD assessment.

### CBF in gray and white matter

CBF values for gray matter were highly correlated with those in white matter across all brain regions (*r* = 0.80, *p <* 0.001). Mean gray matter CBF (65.33 ± 11.1 mL/100 g/min) was significantly higher (*p* < 0.001) than white matter CBF (37.33 ± 5.8 mL/100 g/min) for the whole brain. CBF values were also highly correlated between the left and right hemispheres in both gray matter (*r* = 0.95, *p <* 0.001) and white matter (*r* = 0.94, *p <* 0.001). For simplicity, mean CBF values averaged across the left and right hemispheres are reported, unless hemispheric differences are specifically noted in the correlation analysis between CBF and TCD values.

CBF significantly increased with age across all three flow territories (ACA, MCA, and PCA) in both gray (*p ≤* 0.007) and white (*p ≤* 0.02) matter (Figure 1B; Table 1). For gray matter, CBF increased by 2.4 mL/100 g/min per year in the ACA territory, 2.5 mL/100 g/min per year in the MCA territory, and 1.9 mL/100 g/min per year in the PCA. White matter showed a slightly lower rate of increase: by 1.3 mL/100 g/min per year in the ACA territory, 1.6 mL/100 g/min per year in the MCA territory, and 1.3 mL/100 g/min per year in the PCA (Table 1).

**Table 1:** Pairwise Comparison of CBF across the Three Flow Territories and their Sub-Territories.

			ACA			MCA			PCA		
Gray matter	CBF (mL/100 g/min)		61.24			60.83			78.67		
	CBF/age (mL/100 g/min/year)		2.4			2.5			1.9		
	*p-*values of CBF/age		0.0005			0.0003			0.007		
	*p-*values of difference	ACA				ns			2.2E−11		
		MCA							1.8E−10		
			Proximal	Intermediate	Distal	Proximal	Intermediate	Distal	Proximal	Intermediate	Distal
	CBF (mL/100 g/min)		54.3	65.2	64.3	54.6	65.7	62.2	67.7	81.5	86.9
	*p-*values of difference	Proximal		1.5E−14	3.7E−12		3.1E−12	6.8E−08		5.9E−11	9.2E−09
		Intermediate			ns			9.8E−08			0.009
	*p-*values of difference/age	Proximal		0.03	0.05		0.002	0.0004		0.008	0.02
		Intermediate			ns			ns			ns
White matter	CBF (mL/100 g/min)		41			43.1			56.9		
	CBF/age (mL/100 g/min/year)		1.3			1.6			1.3		
	*p-*values of CBF/age		0.02			0.002			0.02		
	*p-*values of difference	ACA				0.007			1.6E−13		
		MCA							1.3E−10		
			Proximal	Intermediate	Distal	Proximal	Intermediate	Distal	Proximal	Intermediate	Distal
	CBF (mL/100 g/min)		36.4	41.4	45.3	44.4	45.6	39.2	50.5	58.7	61.4
	*p-*values of difference	Proximal		0.0002	2.0E−06		ns	5.4E−05		4.7E−08	2.7E−07
		Intermediate			0.0003			3.9E−11			ns
	*p*-values of difference/age	Proximal		ns	0.03		ns	ns		ns	ns
		Intermediate			0.02			ns			ns

*p-*values were Bonferroni corrected for multiple comparisons; ns = not significant.

### CBF in the arterial flow territories

CBF in the PCA territory was significantly higher than those in the ACA and MCA territories for both gray matter and white matter (*p <* 0.0001). Although white matter CBF differed significantly between the ACA and MCA territories (*p =* 0.007), the corresponding gray matter CBF values did not (Table 1).

Sub-territory CBF values differed significantly across all three arterial flow territories, ACA, MCA, and PCA (Figure 1C; Table 1). Within each territory, CBF in the distal sub-territory was significantly higher than in the proximal sub-territory (*p <* 0.0001) for both gray and white matter. The intermediate sub-territory also showed significantly higher CBF than the proximal sub-territory across all three territories for gray matter (*p <* 0.0001) and in the ACA and PCA for white matter (*p ≤* 0.0002).

Notably, the distal-to-intermediate CBF pattern showed a relative reduction in distal CBF within the MCA territory, but not within the ACA or PCA. In the MCA, distal CBF was significantly lower than intermediate CBF in both gray and white matter (*p* < 0.0001). In the ACA, distal CBF did not differ significantly from intermediate CBF in gray matter but was significantly higher in white matter (*p* = 0.0003). In the PCA, distal CBF was significantly higher than intermediate CBF in gray matter (*p* < 0.0001) and not significantly different in white matter (Table 1).

Age-related effects were also observed for sub-territory CBF differences. The sub-territory difference, (distal − proximal), increased significantly with age in gray matter across all three flow territories (*p =* 0.05 for ACA, *p =* 0.0004 for MCA, and *p =* 0.02 for PCA). Similarly, the (intermediate − proximal) difference increased significantly with age in gray matter (*p =* 0.03 for ACA, *p =* 0.002 for MCA, and *p =* 0.008 for PCA). In white matter, sub-territory differences increased significantly with age only in ACA with *p =* 0.03 for (distal – proximal) and *p =* 0.02 for (distal – intermediate).

### CBF and TCD

**Figure 2:**
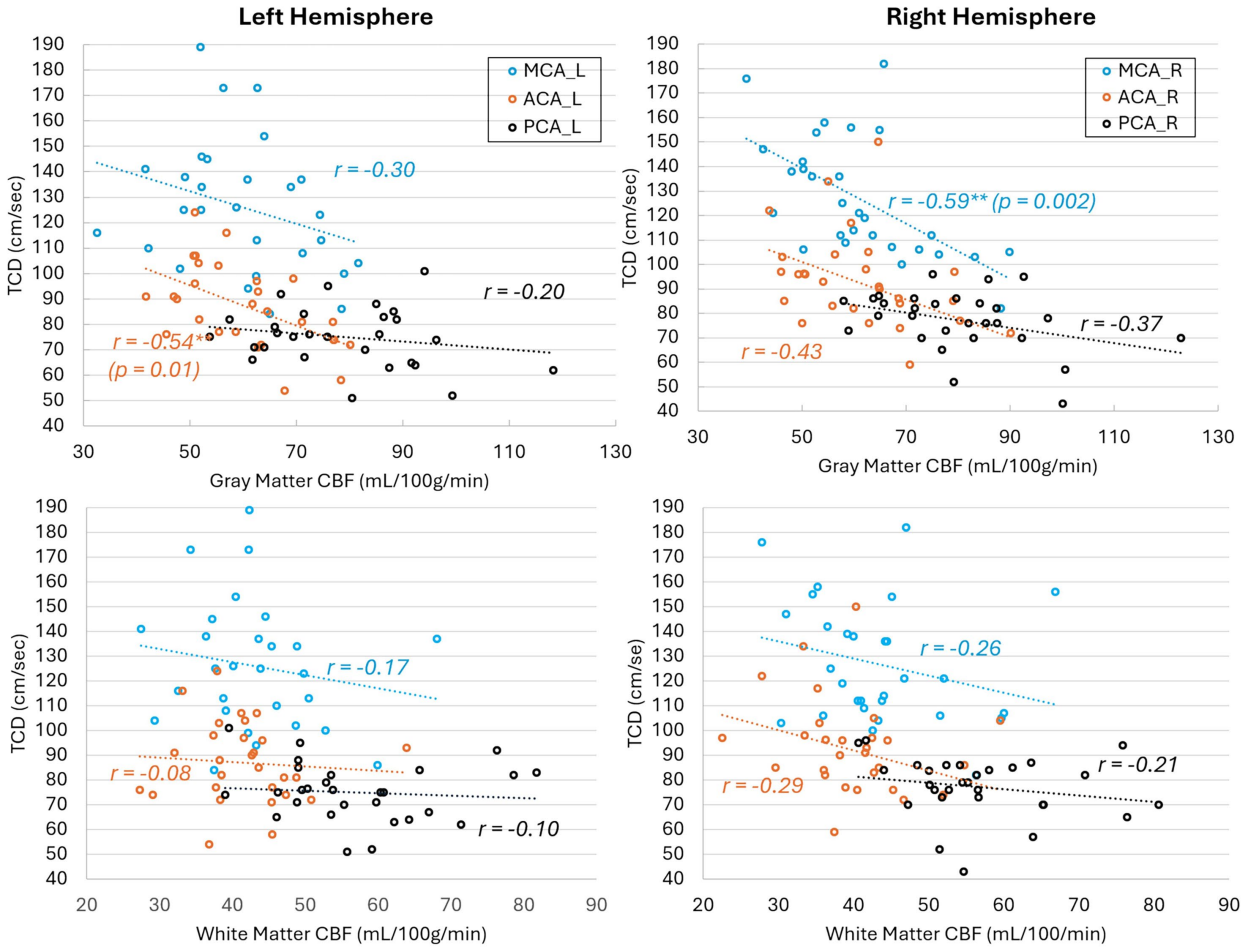
TCD velocity and CBF in corresponding flow territories. In gray matter, CBF and TCD velocity showed a weak inverse correlation only in the left ACA (*r* = −0.54, *p* = 0.01) and right MCA (*r* = −0.59, *p* = 0.002). No significant correlations were observed in the other territories. In white matter, CBF showed no significant correlations with TCD velocity in any of the three territories. *p*-values were Bonferroni corrected. None of the patients had clinically abnormal TCD velocities (≥ 200 cm/s), but five were conditionally high (170–199 cm/s) in the left or right MCA.

Three patients had missing TCD data. Twenty-four (83%) patients had normal TCD velocities bilaterally in the three arteries (MCA, ACA, and PCA). Five patients (17%) had conditionally elevated TCD velocities (170–199 cm/s), with three in the left MCA and two in the right MCA, while their TCD velocities were normal in all other arteries. Of these five patients, four had SCIs detected on brain MRI. The patient with the lowest TCD velocities in the PCA (52 cm/s left, 43 cm/s right) and ACA (54 cm/s left, 59 cm/s right) also had SCIs revealed on MRI, though their TCD values remained within the clinically normal range. One of the two patients who had stenosis by MRA had normal TCD velocities bilaterally in the three arteries, the other had no TCD data available. CBF and TCD velocities in the corresponding arterial territories were not significantly correlated, except in the left ACA and right MCA gray matter, where weak inverse correlations (Akoglu, 2018) were observed. In the ACA gray matter, the correlation was significant in the left hemisphere (*r* = −0.54, *p* = 0.01) but not in the right (*r* = −0.43, *p* > 0.05). In the MCA gray matter, the correlation was significant in the right hemisphere (*r* = −0.59, *p* = 0.002) but not in the left (*r* = −0.30, *p* > 0.05). No significant correlations were observed in the PCA gray matter (left: *r* = −0.20, *p* > 0.05; right: *r* = −0.37, *p* > 0.05). White matter CBF showed no significant correlations with TCD velocity in any of the three territories (Figure 2).

## Discussion

To our knowledge, this is the first study to evaluate sub-territorial differences in cerebral perfusion within major arterial territories in SCA. Our results demonstrate significantly higher CBF values in the distal and intermediate sub-territories compared with the proximal sub-territories of the ACA, MCA, and PCA. Because territorial perfusion mapping identified early hemodynamic alterations that were not captured by global CBF measures or detectable by conventional TCD screening, our work enhanced our understanding of hemodynamic adaptations in children with SCA and highlighted the vulnerability of distal microcirculation. These findings have important implications for prognostication, treatment decision-making, and evaluation of therapeutic effects in this high-risk population.

Consistent with lobar patterns observed in healthy children (Zou et al., 2021), who show the highest CBF in the occipital lobes, children with SCA in our study had the highest CBF values in the PCA territory, which primarily supplies the occipital lobes. Similar to previous reports (Stinnett et al., 2025; Jordan et al., 2025), CBF increased significantly with age in children with SCA, opposite to the age-related decline seen in healthy children (Zou et al., 2021; Avants et al., 2015). This trend suggested a progressive compensatory response to chronic anemia as children with SCA grow older (Stinnett et al., 2025; Jordan et al., 2025). Moreover, the higher CBF observed in distal and intermediate sub-territories compared with proximal sub-territory within each flow territory may represent a unique feature of SCA, as these sub-territory differences also increased with age, further supporting the progressive nature of this compensatory adaptation.

A notable finding of this study was that distal CBF in the MCA territory was significantly lower than intermediate CBF, a pattern not observed in the ACA or PCA territories. This relative reduction in distal MCA perfusion may highlight a particular vulnerability of this region to ischemic injury in children with SCA. The MCA territory supplies posterolateral parts of the frontal lobe and posterior-lateral part of the parietal lobe, including the deep white matter and border-zone regions between the ACA and PCA distributions. In the context of chronic anemia and elevated compensatory CBF, these distal MCA regions may reach the limits of autoregulatory vasodilation, making them more susceptible to hemodynamic stress and oxygen delivery failure. The SCI locations observed in our patients were predominantly within the MCA territory, particularly in the distal border-zone regions involving the deep white matter of the frontal and parietal lobes, consistent with common SCI locations reported from previous studies (DeBaun et al., 2012). Thus, the lower distal CBF in MCA in this study may reflect early hemodynamic strain and provide a physiological explanation for the predilection of the MCA territory for SCI in SCA.

Since the ACA, MCA, and PCA should capture most of the incoming blood flow proximally, it seems counterintuitive that distal sub-territories showed higher CBF than proximal regions. If we assume blood volume does not change, then increased CBF signifies a decrease in mean transit time in the capillaries. In this scenario, fast-moving blood through capillaries without sufficient time for oxygen extraction could cause distal ischemia. Alternatively, if we do not assume blood volume is constant, then the distal territories would receive extra blood from extracranial collaterals. This would raise both CBF and blood volume without necessarily shortening the transit time. Either way, the higher CBF observed in distal sub-territories may reflect compensatory hemodynamic adjustments in children with SCA. This finding suggests the compensatory increase in CBF occurs first at the microvascular level in the distal regions, in response to local tissue oxygen demand. This pattern is consistent with the fact that silent cerebral infarcts tend to occur at vascular border zones (watershed regions) (Fields et al., 2019), also “distal” in circulation.

This finding is also consistent with the conceptualization of SCA as a small-vessel disease (Sundd et al., 2019; Stotesbury et al., 2019), in which microvascular dysfunction and vascular remodeling contribute to cerebral injury. Notably, although none of the participants demonstrated abnormally high TCD velocities and only 17% had conditionally elevated values, SCIs were present in 38% of patients, highlighting the vulnerability of the distal microcirculation even in the absence of overt large-artery disease. We observed SCIs in patients with both conditional TCD velocities and relatively low TCD velocities, as well as stenosis in a patient with normal TCD. However, given the small number of affected patients, the clinical significance of these observations cannot be determined from the present study. Nevertheless, these findings suggest that clinically normal or conditional TCD velocities may not fully capture underlying cerebrovascular injury. A critical question concerning cerebral hemodynamics is when does elevated CBF reflect impending neurological insult as opposed to stable compensation. Elevated TCD velocity may indicate that the compensatory capacity achieved through increases in vessel numbers or compliance has reached its limit. Beyond this point, CBF can be only maintained by increasing blood velocity. A compensated intracranial vasculature that is dilated and compliant might be more dependent on adequate perfusion pressure to maintain flow. In the setting of decreased perfusion pressure due to sepsis, dehydration, or decreased cardiac output, deep white matter watershed infarcts may occur. The distinction between flow-limiting stenoses and inadequate cerebral perfusion pressure has implications for the etiology of stroke in individuals with SCA.

TCD velocity and ASL-derived CBF were not positively correlated in our study. This is largely because the two modalities measure different aspects of cerebral hemodynamics. ASL quantifies tissue-level perfusion (mL/100 g/min), whereas TCD assesses blood flow velocity within large arteries (cm/s) (Purkayastha and Sorond, 2012). Without any adjustment, they are not directly correlated with each other (Croal et al., 2017). With appropriate adjustments for tissue mass and vessel cross-sectional area, TCD-derived flow estimates can be positively correlated with ASL-derived CBF, but this correlation tends to be weaker in children with SCA than healthy children (Croal et al., 2017). Elevated TCD velocity may represent proximal arterial stenosis in some patients with SCA (Seibert et al., 1998), but more broadly it represents a hyperemic compensatory state with increased overall CBF (Ausavarungnirun et al., 2006). However, not all arterial stenoses produce abnormal TCD velocities, as demonstrated by one patient in our cohort with significant stenosis but normal TCD findings. Additionally, TCD-measured blood flow velocity reflects not only volumetric blood flow but also multiple aspects of vascular physiology and pathology, including vessel caliber, endothelial dysfunction, vascular remodeling, vessel wall thickening, and hemodynamic factors such as shear stress. These mechanisms are particularly relevant in sickle cell disease and may contribute to the observed variability in TCD velocities. Thus, the correlation between TCD velocity and tissue perfusion remains complex, especially in patients with SCA. A possible explanation for the observed weak inverse relationship between TCD velocity and CBF involves collateral vessel recruitment or a reduction in intravascular resistance in response to regional oxygen supply–demand mismatch. Increased CBF may result from the recruitment of parallel microvascular pathways that reduce overall vascular resistance and enhance tissue perfusion, while simultaneously lowering the velocity in any single feeding artery.

One important limitation of this study is its relatively small sample size. Therefore our findings should be interpreted with appropriate caution. The other limitation is the narrow range of TCD velocities observed among participants, none of whom with abnormal TCD velocities. The restricted TCD range may have reduced our ability to detect stronger associations between TCD velocity and CBF, particularly at higher velocity thresholds typically observed in more severe vasculopathy cases. Consequently, our findings may not be directly applicable to patients with abnormal TCD values. Future studies with larger and more heterogeneous cohorts, including patients with a wider range of TCD velocities and clinical severity, are needed to validate these results and further clarify the interplay between macrovascular flow velocity and tissue-level perfusion.

Despite these limitations, the unique sub-territorial patterns of CBF observed in children with SCA provide valuable insights into cerebrovascular physiology in this population. These findings show that regional increase in perfusion can be detected even in the absence of elevated TCD velocities, indicating that alterations in microvascular flow and autoregulatory adaptation may occur early in the disease process. Conversely, sub-territory CBF mapping may also serve as a sensitive biomarker to detect early hemodynamic changes following treatment with emerging pharmacologic agents (Brothers et al., 2024; Heitzer et al., 2024) or curative gene therapies (Sharma et al., 2025). However, this study should be considered exploratory. While TCD remains the standard screening tool for stroke risk assessment in pediatric sickle cell disease, territory-specific ASL MRI offers additional information regarding regional tissue perfusion. Our findings suggest that ASL and TCD provide complementary rather than interchangeable measures of cerebrovascular status. Further studies in larger cohorts, particularly including patients with abnormal TCD velocities, are required to determine the potential clinical utility of ASL MRI in routine monitoring and risk assessment.

In conclusion, our findings suggest that compensatory increases in CBF in response to chronic anemia occur first primarily at the microvascular level within the distal and intermediate sub-territories, underscoring the vulnerability of the distal microcirculation. The relative reduction in distal MCA perfusion compared with the intermediate region may help explain the greater susceptibility of this area to ischemic injury and silent cerebral infarction in children with SCA. By mapping CBF across arterial territories and sub-territories, this study provides new insights into cerebrovascular compensation in pediatric SCA, demonstrates the limitations of relying solely on global CBF measures, and highlights the value of territorial perfusion imaging for detecting early microvascular compromise and treatment-related vascular changes.

## Data Availability

The raw data supporting the conclusions of this article will be made available by the authors, without undue reservation.

## Footnotes

1
https://www.fil.ion.ucl.ac.uk/spm/
